# Patients with endometriosis may experience worse clinical manifestations and therapeutic outcomes during COVID-19 in western China- a case series comparative analysis

**DOI:** 10.1186/s12905-023-02344-w

**Published:** 2023-04-28

**Authors:** Sanhong Liu, Cong Hou, Sisi Tang, Shutong Bai, Ying Deng

**Affiliations:** 1 Department of Prevention, Chongqing Traditional Chinese Medicine Hospital, Chongqing, 400021 People’s Republic of China; 2 Department of Gynecology, Chongqing Traditional Chinese Medicine Hospital, No. 6, Panxi 7 Branch Road, Jiangbei District, Chongqing, 400021 People’s Republic of China; 3 Chongqing Key Laboratory of Traditional Chinese Medicine to Prevent and Treat Autoimmune Diseases, Chongqing Traditional Chinese Medicine Hospital, Chongqing, 400021 People’s Republic of China

**Keywords:** Endometriosis, COVID-19, Treatment outcome, Psychological scores, The quality of life

## Abstract

**Background:**

Endometriosis is a crippling, ongoing, chronic inflammatory condition. The management of these patients has been impacted by the current COVID-19 pandemic, which is still controversial. This study compared the clinical therapy outcomes and psychological scores between before and during- the epidemic.

**Method:**

The data of patients who were diagnosed with endometriosis in the Department of Gynecology, Chongqing Traditional Chinese Medicine Hospital from January 2018 to December 2022 were collected. The patients were divided into pre- and intra-COVID groups. The treatment results and psychological status of the two groups were compared.

**Results:**

A total of 1022 patients with endometriosis were enrolled, with a mean age of 33.16 ± 9.81 years and a BMI of 23.90 ± 3.04 kg/m^2^, of which 434 cases (434/1022, 42.5%) were in the pre-COVID group and 588 cases (588/1022, 57.5%) in the intra-COVID group. Both groups were well balanced for age, BMI, history of abdominopelvic surgery, family relationships, education level, and duration between initial diagnosis and admission. Compared to the Pre-COVID group, the intra-COVID group had a higher proportion of patients with chronic pelvic pain (297/434, 68.4% vs. 447/588, 76.0%, p = 0.007) and dysmenorrhea (249/434, 62.8% vs. 402/588, 70.0%, p < 0.001), more patients requiring surgery (93/434, 21.4% vs. 178/588, 30.3%, p = 0.002) and longer hospital stays (5.82 ± 2.24 days vs. 7.71 ± 2.15 days, p < 0.001). A total of 830 questionnaires were completed. In the Intra-COVID group, PHQ-2 (2 (2, 3) vs. 3 (2,4), p < 0.001), GAD-2 (2 (1, 2) vs. 3 (2, 3), p < 0.001), PHQ-4 (4 (3, 5) vs. 5 (4, 7), EHP-5 (20.26 ± 6.05 vs. 28.08 ± 7.95, p < 0.001) scores were higher than that in the pre-COVID group, while BRS (3.0 (2.2, 4.0) vs. 2.4 (1.8, 3.8), p = 0.470) were not significantly different.

**Conclusion:**

During the COVID-19 epidemic, patients with endometriosis may have reduced visits to the hospital, more severe related symptoms, longer length of hospital stays, and worse quality of life, with the possible cause being a disturbance in hormone levels through increased anxiety and depression. This provides a valid clinical basis for optimizing the management of patients with endometriosis and for early psychological intervention during the epidemic.

## Introduction

Endometriosis (endometriosis) is one of the most common gynecological conditions, affecting up to 10% of women of childbearing age [1]. It is a chronic inflammatory response to the presence, growth, infiltration, recurrent bleeding, and tissue repair of endometrial tissue (glands and mesenchyme) outside the uterus. Endometriosis affects 71–87% of women with persistent pelvic discomfort and 20–50% of women with infertility [2]. The pathogenesis of endometriosis has not been fully investigated and may be related to sex hormones, immunity, inflammation, genetics, abnormal intestinal permeability, and hosts’ microbiome profiles [3–5]. It has atypical and non-specific clinical symptoms, such as chronic pelvic pain, infertility, and masses, and is often not diagnosed and treated promptly [6, 7]. Treatment of endometriosis, particularly deep infiltrating endometriosis, is often challenging and should be determined by the patient’s medical history, stage of disease, severity of symptoms, and personal choice. Conservative treatment is the mainstay based on a comprehensive multidisciplinary assessment, with surgery only performed if necessary [8]. Therefore, endometriosis seriously affects women’s daily life, sexuality, Intestinal function, urinary tract function, and even their psychological health [9–12].

The current coronavirus disease 2019 (COVID-19) pandemic has significant effects on the diagnosis, management, and follow-up of patients with chronic conditions and is placing tremendous strain on health systems around the world [13]. During the COVID-19 pandemic, some minor, non-emergency, or non-essential patients were restricted because of the blockade measures taken to prevent the spread of the virus. Similarly, intermittent blocking in Chongqing in southwest China may harm those with chronic illnesses like endometriosis. The continuity of the diagnosis and treatment follow-up is essential to improve endometriosis patients’ prognosis and quality of life [14]. In addition, some studies have shown that hormone therapy in patients with endometriosis might increase the rate and severity of COVID-19 infections [15], as well as delay surgical treatment due to the focus on covid-19 patients [16].

To study the impact of the COVID-19 pandemic on patients with endometriosis, we retrospectively collected clinical data at our hospital before and after the pandemic, comparing chronic pain, psychological status, and quality of life related to endometriosis, thus providing a basis for improving the outcome of the patient’s prognosis.

## Method

### Patient selection

The database of the *Chongqing Traditional Chinese Medicine Hospital* was searched retrospectively. Patients diagnosed with endometriosis from January 2018 to December 2022 were collected. They were diagnosed according to the criteria of the Guideline for the diagnosis and treatment of endometriosis (Third edition) [2]. The diagnostic criteria are as follows: (1) Clinical manifestation: dysmenorrhoea, affecting daily activities and life, chronic pelvic pain, painful intercourse, or post-coital pain, accompanied by profuse menstrual flow or menostaxis. (2) Gynecological examination: posteriorly tilted and fixed uterus, poorly mobile cystic masses in the adnexa, painful nodules in the posterior vaginal vault; (3) Auxiliary examination: CA125 increased, ultrasound test reveals a round uniform, densely dotted echogenicity, or nodular irregular hypoechogenicity. This study was reviewed and approved by the ethics committee of Chongqing Traditional Chinese Medicine Hospital, approval number: 2022-DWSY-BST. All patients had signed a written informed consent form before treatment. The study was performed following the Declaration of Helsinki. Patients who met the following criteria were included: (1) Aged between 18 and 45 years old. (2) In compliance with the above diagnosis criteria of endometriosis. (3) No hormonal or other treatment in the 3 months prior to admission. Patients who met the following criteria were excluded: (1) In menopause; (2) Suffering from severe liver and kidney dysfunction, cardiovascular disease, hematological system diseases, and endocrine disorders; (3) Combined with other gynecological diseases, such as uterine fibroids, adenomyosis, pelvic infections, pelvic malignancies; (4) Comorbid psychiatric disorders; (5) refused to be followed up. Data regarding age, BMI (Body Mass Index), surgical history, Marital Status, family relations, educational level, time since diagnosis, Chronic pelvic pain score, dysmenorrhea, CA125 level, whether to perform surgery, r-ASRM score, operative time, operative blood loss, length of hospital stay, PHQ-4 score, BRS score, EHP-5 score, were collected. Pain intensity was assessed via the visual analog pain scale (VAS), a continuous scale ranging from 0 to 10 (10 being the most potent imaginable pain) [17].

The Patient Health Questionnaire for Depression and Anxiety (PHQ-4) instrument was used to assess the psychological burden of the study group [18]. Two items from the Patient Health Questionnaire for Depression (PHQ-2) and the Generalized Anxiety Disorder Scale (GAD-2) were combined to form the PHQ-4. PHQ-4 is a general depression and anxiety assessment instrument. The impact of resilience on the outcomes of mental health was evaluated using the Brief Resilience Scale (BRS) [19]. The Endometriosis Health Profile-5 (EHP-5) was used to assess the quality of life of these patients [20].

### Treatment options

The main treatment options for patients with endometriosis are medication and surgery. Although endometriosis cannot be cured, effective and secure treatment must be continued until menopause or planned pregnancy. patients who plan to have children should continue to resume long-term management of medication as soon as possible after the completion of pregnancy [21]. There are five main types of medication: non-steroidal anti-inflammatory drugs (NSAIDs), progestins, combined oral contraceptives (COCs), gonadotropin-releasing hormone agonists (GnRH-a), and Chinese herbal medicine.

The three main surgical procedures are hysterectomy, hysterectomy with bilateral adnexa, and simple focal resection to retain the patient’s reproductive function. Adequate assessment and preparation prior to surgery to fully perspective the possible risks of urological and intestinal injury, or the possibility of multidisciplinary treatment (MDT) for patients with complex conditions to develop an appropriate surgical plan. Medication is kept up after surgery until the patient meets the requirements for discharge.

### Statistical analysis

Data analysis was performed using SPSS 26.0 software (Statistical Package for the Social Sciences, Chicago, IL, USA), and p < 0.05 was considered a significant difference. The classification variables were represented as frequency and percentage, while the data for continuous variables were represented as mean standard deviation (SD). Categorical variables were expressed as frequencies and percentages (%), and the two study groups were compared using the standard chi-square test or Fisher’s exact test. Continuous variables that did not follow a normal distribution were expressed as the median, the 25th (Q1) quantile, and the 75th (Q3) quantile, and were compared using the Wilcoxon rank sum test. Data for continuous variables that followed a normal distribution were expressed as mean standard deviation using the independent samples t-test or corrected t-test.

## Result

### Patients’ clinic and demographic characteristics

From January 2018 to December 2022, we included a total of 1022 patients with endometriosis, with a mean age of 33.16 ± 9.81 years and a BMI of 23.90 ± 3.04 kg/m^2^. Patients with no previous history of pelvic surgery (94.9% vs. 95.4, p = 0.724), harmonious family relationships (87.1% vs. 83.7%, p = 0.128) and a high level of education (74.2% vs. 77.9%, p = 0.169) were the majority in both groups. There were 434 cases (434/1022, 42.5%) in the pre-COVID group and 588 cases (588/1022, 57.5%) in the intra-COVID group. The baseline clinical characteristics of all cases were shown in Table 1.

**Table 1:** The baseline clinical characteristics and treatment outcomes of all cases

Variables	Total (%)	Pre-COVID (%)	Intra-COVID (%)	x^2^/t	p-Value
**Patients (n)**	1022 (100.0%)	434 (42.5%)	588 (57.5%)		
**Age (years)**	33.16 ± 9.81	32.55 ± 9.28	33.85 ± 10.51	1.709	0.088
**BMI (kg/m** ^**2**^ **)**	23.90 ± 3.04	22.80 ± 3.02	23.01 ± 3.06	0.995	0.320
**Surgical history**				0.125	0.724
Absent	973 (95.2%)	412 (94.9%)	561 (95.4%)		
Present	49 (4.8%)	22 (5.1%)	27 (4.6%)		
**Been married**	635 (62.1%)	283 (65.2%)	352 (59.9%)	3.030	0.082
**Harmonious family relations**	870 (85.1%)	378 (87.1%)	492 (83.7%)	2.311	0.128
**Educational level**				1.889	0.169
Bachelor’s degree or above	780 (76.3%)	322 (74.2%)	458 (77.9%)		
Lack of education	242 (23.7%)	112 (25.8%)	130 (22.1%)		
**Time since diagnosis (years)**	2.83 ± 1.13	2.87 ± 1.18	2.78 ± 1.09	0.626	0.532
**Chronic pelvic pain**				7.259	0.007
Absent	278 (27.2%)	137 (31.6%)	141 (24.0%)		
Present	744 (72.8%)	297 (68.4%)	447 (76.0%)		
**Chronic pain score**	3.72 ± 1.38	3.29 ± 1.24	4.31 ± 1.41	10.232	< 0.001
**Dysmenorrhea**				13.052	< 0.001
Absent	277 (33.8%)	185 (37.2%)	186 (30.0%)		
Present	542 (66.2%)	249 (62.8%)	402 (70.0%)		
**CA125**	18.86 ± 6.31	17.84 ± 5.79	20.03 ± 6.52	2.659	0.008
**Surgery**	271 (26.5%)	93 (21.4%)	178 (30.3%)	10.022	0.002
**Length of hospital stay (days)**	6.75 ± 2.17	5.82 ± 2.24	7.71 ± 2.15	6.025	< 0.001

Data were expressed as mean ± standard deviation or the number of cases (%). BMI, body mass index

**Fig. 1:**
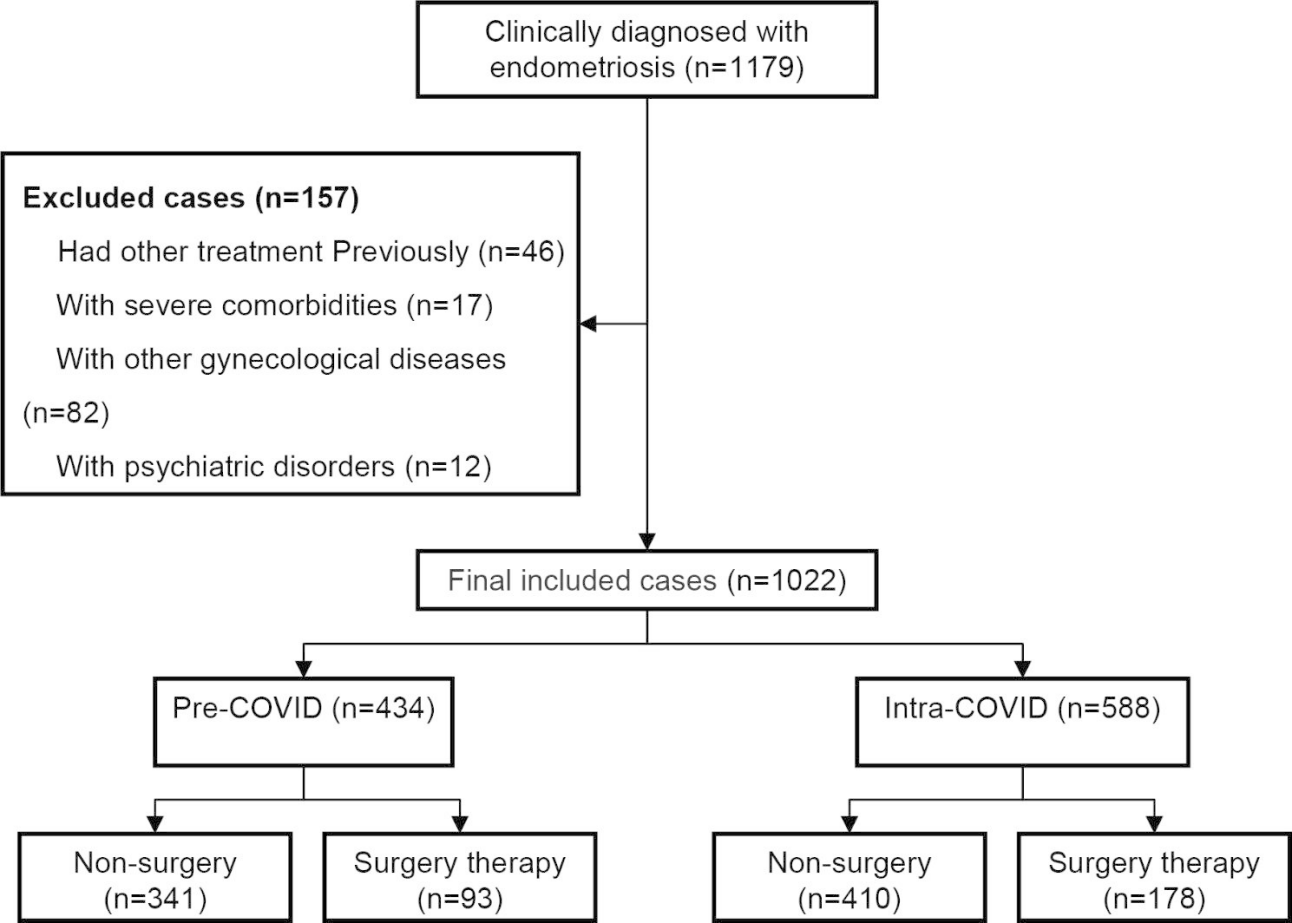
Flowchart summarizing the patient selection

From December 2019 to the present, the COVID-19 outbreak has persisted, with intermittent outbreaks followed by isolation and quarantine. In Chongqing, China, more extensive isolation and quarantine took place in November 2022, while from 5 December the society was liberalized. Compared to the pre-COVID period, the number of patients admitted to the hospital for endometriosis fluctuated significantly monthly during the epidemic. Interestingly, in November and December 2022, the number of patients was almost zero (Fig. 1).

**Fig. 2:**
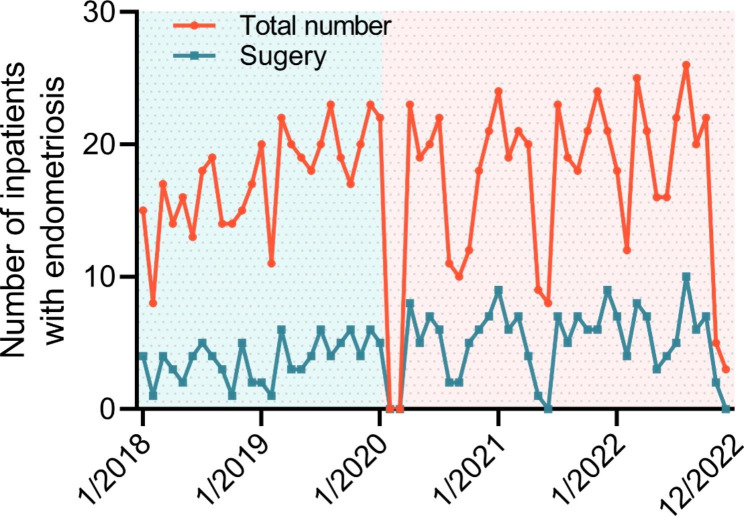
All inpatients and surgeries with endometriosis monthly. The bluish box represents the pre-COVID-19 period and the pink box indicates the COVID-19 period.

There were no significant differences between the two groups in terms of age, BMI, history of abdominopelvic surgery, family relationships, education level, and duration between initial diagnosis and admission. Compared to the Pre-COVID group, the intra-COVID group had a higher proportion of patients with chronic pelvic pain (297/434, 68.4% vs. 447/588, 76.0%, p = 0.007) and dysmenorrhea (249/434, 62.8% vs. 402/588, 70.0%, p < 0.001), higher CA125 (17.84 ± 5.79 vs. 20.03 ± 6.52, p = 0.008), more patients requiring surgery (93/434, 21.4% vs. 178/588, 30.3%, p = 0.002) and longer hospital stays (5.82 ± 2.24 days vs. 7.71 ± 2.15 days, p < 0.001) (Table 1). Patients in the intra-COVID group also had higher levels of chronic pelvic pain (3.29 ± 1.24 vs. 4.31 ± 1.41, p < 0.001) and dysmenorrhea (5.22 ± 1.43 vs. 6.41 ± 1.58, p < 0.001), Fig. 2.

### Analysis of surgical patients

**Fig. 3:**
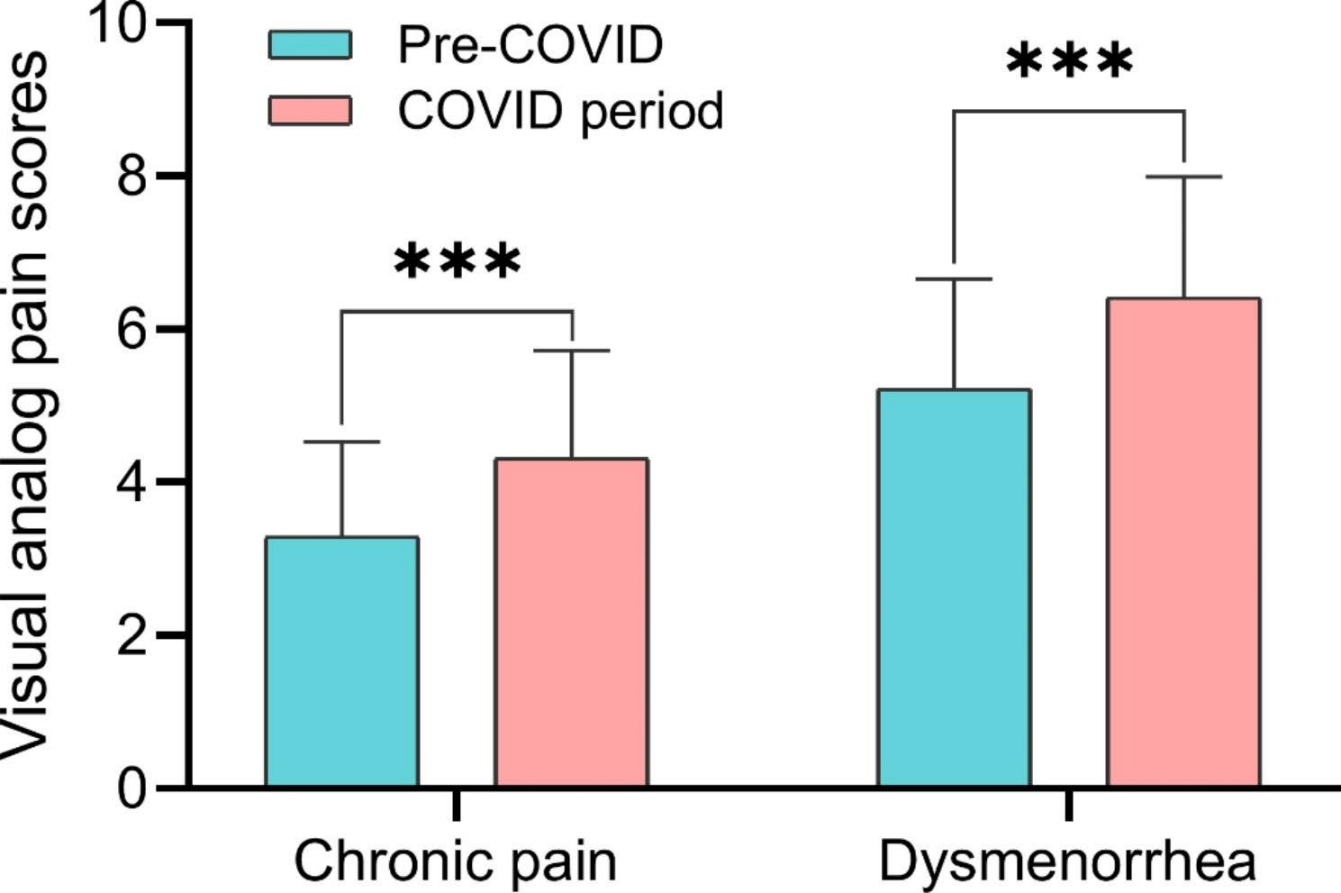
Visual analog pain scores of patients with endometriosis. Chronic pain represents patients’ chronic pelvic pain. *** indicates a significant difference with p < 0.001.

We further analyzed patients with endometriosis who underwent surgery. There were no significant differences between the two groups in terms of age, BMI, history of abdominopelvic surgery, family relationships, education level, duration between initial diagnosis and admission, r-ASRM score, operative time, and intraoperative blood loss. Compared to the pre-COVID group, the intra-COVID group had a higher proportion of patients with chronic pelvic pain (62/93, 66.7% vs. 149/178, 83.7, p = 0.001) and dysmenorrhoea (40/93, 43.0% vs. 121/178, 68.0%, p < 0.001) (Fig. 3), a higher CA125 level (25.13 ± 6.59 vs. 31.11 ± 9.49, p = 0.002) and longer length of hospital stay (6.67 ± 1.64 vs. 8.60 ± 2.66, p < 0.001) (Table 2).

**Table 2:** Subgroup analysis of surgical patients before and during the COVID-19 epidemic

Variables	Total (%)	Pre-COVID (%)	Intra-COVID (%)	x^2^/t	p-Value
**Patients (n)**	271 (26.5%)	93 (21.4%)	178 (30.3%)		
**Age (years)**	33.30 ± 9.92	32.56 ± 9.87	33.90 ± 9.95	0.978	0.333
**BMI (kg/m** ^**2**^ **)**	22.71 ± 2.86	22.85 ± 2.96	22.60 ± 2.77	0.638	0.524
**Surgical history**				0.118	0.731
Absent	247 (91.1%)	84 (90.3%)	163 (90.8%)		
Present	24 (8.9%)	9 (9.7%)	15 (9.2%)		
**Been married**	161 (59.4%)	54 (58.1%)	107 (60.1%)	0.106	0.744
**Harmonious family relations**	227 (83.8%)	76 (81.7%)	151 (84.8%)	0.435	0.510
**Educational level**				0.805	0.370
Bachelor’s degree or above	173 (63.8%)	56 (60.2%)	117 (65.7%)		
Lack of education	98 (36.2%)	37 (39.8%)	61 (34.3%)		
**Time since diagnosis (years)**	3.01 ± 1.96	3.23 ± 2.26	2.83 ± 1.66	1.417	0.158
**Chronic pelvic pain**				10.291	0.001
Absent	49 (23.7%)	31 (33.3%)	29 (16.3%)		
Present	158 (76.3%)	62 (66.7%)	149 (83.7%)		
**Chronic pain score**	4.13 ± 1.35	3.81 ± 1.31	4.79 ± 1.47	7.287	< 0.001
**Dysmenorrhea**				15.790	< 0.001
Absent	110 (40.6%)	53 (57.0%)	57 (32.0%)		
Present	161 (59.4%)	40 (43.0%)	121 (68.0%)		
**CA125 (U/ml)**	28.45 ± 7.68	25.13 ± 6.59	31.11 ± 9.49	3.132	0.002
**r-ASRM**				0.155	0.693
I-II	203 (74.9%)	71 (76.3%)	132 (74.2%)		
III-IV	68 (25.1%)	22 (23.7%)	46 (25.8%)		
**Operative time (min)**	108.45 ± 33.86	115.91 ± 41.84	106.25 ± 26.27	0.433	0.665
**Operative blood loss (ml)**	85.60 ± 20.13	82.47 ± 12.74	88.16 ± 27.09	0.507	0.613
**Length of hospital stay (days)**	7.78 ± 2.05	6.67 ± 1.64	8.60 ± 2.66	4.026	< 0.001

Data were expressed as mean ± standard deviation or the number of cases (%). BMI, body mass index. R-ASRM revised American Society for Reproductive Medicine score

### Analysis of mental health

A total of 830 questionnaires were completed, with 358 (82.5%) from the pre-COVID group and 472 (80.3%) from the intra-COVID group. In the Intra-COVID group, PHQ-2 (2 (2, 3) vs. 3 (2,4), p < 0.001), GAD-2 (2 (1, 2) vs. 3 (2, 3), p < 0.001), PHQ-4 (4 (3, 5) vs. 5 (4, 7), EHP-5 (20.26 ± 6.05 vs. 28.08 ± 7.95, p < 0.001) scores were higher than that in the pre-COVID group, while BRS (3.0 (2.2, 4.0) vs. 2.4 (1.8, 3.8), p = 0.470) were not significantly different (Table 3).

**Table 3:** Comparative analysis of the phycological status and quality of life of two groups of patients with endometriosis before and during the COVID-19 epidemic

Variables	Pre-COVID (%)	Intra-COVID (%)	Z/t	p-Value
**Patients (n)**	358 (82.5%)	472 (80.3%)		
**PHQ-2**	2 (2, 3)	3 (2, 4)	10.376	< 0.001
**GAD-2**	2 (1, 2)	3 (2, 3)	4.762	< 0.001
**PHQ-4**	4 (3, 5)	5 (4, 7)	13.161	< 0.001
**BRS**	3.0 (2.2, 4.0)	2.4 (1.8, 3.8)	0.722	0.470
**EHP-5**	20.26 ± 6.05	28.08 ± 7.95	16.083	< 0.001

PHQ-1, the Patient Health Questionnaire for Depression. GAD-2, the Generalized Anxiety Disorder Scale. PHQ-4, the Patient Health Questionnaire for Depression and Anxiety. BRS, the Brief Resilience Scale. EHP-5, The Endometriosis Health Profile-5

## Discussion

This study is a comparative study of the clinical presentation and mental health of endometriosis before and during the COVID-19 epidemic. Our study showed that during the COVID-19 epidemic, there was a significant decrease in the number of patients presenting to our department with endometriosis, and with isolation and containment, the number of patients was more volatile and more severe. In addition, they had more severe clinical symptoms such as chronic pelvic pain and dysmenorrhea, upregulated CA125 levels, and poorer psychosocial status. This study would give a clinical basis for patients with endometriosis to monitor the course of the disease, improve their psychological state and alleviate the effects of endometriosis through self-regulation or telemedicine during the special period of the epidemic.

The pathogenesis of endometriosis is unclear. The dominant theory is that it is caused by retrograde menstruation, a process in which menstrual blood containing endometrial cells flows backward through the fallopian tubes and into the pelvic cavity instead of being expelled from the body [22]. Some hypotheses postulate that genetic, immunological, hormonal, or environmental variables may all play a role in the development of endometriosis [3–5]. The treatment of endometriosis is based on conservative measures [8]. The fundamental principle of surgical treatment is to remove the lesion, preserve as much healthy tissue as possible and protect the function of the pelvic organs to reduce pain, reduce the recurrence rate and improve the quality of life [23].COVID-19 has largely affected people’s access to health care. In South Korea, the number of visits by stroke patients fell by 1/3 during the COVID-19 epidemic and the proportion of patients with TIA also fell significantly (2.91–9.97%) [24]. One study showed a 46% drop in new cancer patients in the US between March and April 2020 [25]. Our study showed a 9.68% decrease in the average monthly number of patients with endometriosis during the epidemic, which is in line with our common perception [26]. The probable reason for this is that the isolation to avoid the spread of the virus during the epidemic could largely discourage patients from seeking medical care. Current studies of endometriosis have not found a significant decrease in its incidence, which is around 100–300 per 100,000 per year, with a prevalence of around 5% [27]. Also, our study found a significant increase in the proportion of patients operated on for endometriosis during the epidemic, although their absolute numbers did not increase. Medication is the mainstay of endometriosis treatment, while surgery is not the primary option [2]. Therefore, the increase in the proportion of surgery during the epidemic may be due to the followings: (1) the reduction in the total number of patients with endometriosis; (2) patients with common mild symptoms may prefer to take their own oral medication to relieve their symptoms rather than opting to visit the hospital; (3) the indication for surgery was reached due to the more rapid and severe progression of endometriosis during the COVID epidemic.

Previous research had found that low level of education was a risk factor for the development of psychological disorders and that higher level of education may increase people’s cognitive level and broaden the dimension of thought, thus empowering them with more coping mechanisms for stress, leading to more favorable psychological outcomes [28]. In contrast, our study showed that the psychological-related scores were significantly higher in the intra-COVID group than in the pre-COVID group, while there was no significant difference between the educational levels. This may be due to bias between study selection samples. In addition, a study by W. Y. Low et al. pointed out that patients with endometriosis who had higher socioeconomic status and a higher level of education were more likely to have adverse symptoms such as stress, dysmenorrhea, and prolonged pain [29]. A Gosset et al.‘s study of factors associated with quality of life in patients with deep infiltrative endometriosis treated by bilateral oophorectomy showed that smoking and being overweight was associated with quality of life in patients with endometriosis, rather than education level [30]. These results may suggest that significant social stress may override the effects of other factors on patients with endometriosis.

Our study also found that during the epidemic, an increased proportion of patients with endometriosis presented with chronic pelvic pain, dysmenorrhea, and more severe pain. The study by Vojka Lebar et al. noted that changes in menstrual volume and menstrual cycle length were a consequence of SARS-CoV-2 infection, but that the severity of COVID-19 was not associated with menstrual cycle changes [31]. Moreover, analysis at the psychological level showed that this group of patients had more problems with depression and anxiety, and their quality of life was severely affected. We all know that depression-related psychological problems, pelvic pain, dysmenorrhea, and difficulties with sexual intercourse are related causes of quality of life for people with endometriosis [32]. Their underlying driving mechanism may be that mental health problems cause hormonal disturbances in women, which can lead to an increase in the clinical symptoms of endometriosis and even surgical intervention. Of course, the pathogenesis of endometriosis is yet to be further investigated.

We conducted a subgroup analysis for surgically treated endometriosis, and it showed that there was no difference in r-ASRM degree, duration of surgery, and blood loss. The r-ASRM score is associated with several factors and reflects the clinical severity of endometriosis [33]. The duration of the operation, blood loss, and the difficulty of the operation are determined by the history of previous surgical pelvic laparotomy and r-ASRM score. Our study suggests that the epidemic did not increase the severity of endometriosis, or was not severe enough to have affected the r-ASRM score.

Social isolation and quarantine may have a greater impact on human mental health. During the SARS-CoV-1 epidemic in Canada, isolation for more than 10 days significantly impaired people’s psychological wellness [34]. During COVID-19, social network limitations were shown to be an independent risk factor for the development of generalized anxiety, and social connectedness increased well-being and life satisfaction [35]. Previous studies had also shown that endometriosis and its associated symptoms were strongly associated with a decline in mental health [36]. On the other hand, patients were more inclined to express their negative emotions due to the control. Therefore, rational psychological regulation and a greater sense of self-identity are essential during the epidemic to minimize the signs of endometriosis and slow down its development [37]. In our study, psychological scores, and quality of life scores of patients with endometriosis were generally higher during the epidemic, which is further evidence that social stress may exacerbate endometriosis.

There were also limitations to this study. Firstly, this study only analyzed patients with endometriosis who were hospitalized. Endometriosis is a long-term, chronic condition and most patients do not require hospitalization through self-regulation or pharmacological treatment [2]. Therefore, our study may have a narrow coverage and does not reflect the reality of a larger group of patients with endometriosis. Secondly, the surgical patients we included were performed by three surgeons, which may have had an impact on surgical outcomes. However, they were all senior and experienced in gynecological surgery, which ensured consistency among surgical patients. Thirdly, this study only provided a clinically relevant analysis of endometriosis, without an in-depth mechanistic analysis. The mechanisms underlying the pathogenesis and severity of endometriosis are complex and may be related to the peritoneal microenvironment changes caused by immune cells, inflammatory responses, etc. [4]. Lastly, we may have had a selective bias in this study since we only studied inpatients and excluded adenomyosis.

## Conclusion

The COVID-19 epidemic may have reduced visits to patients with endometriosis, increased their symptoms, severity, length of hospital stays, and reduced quality of life, with the possible cause being a disturbance in hormone levels through increased anxiety and depression. This provides a valid clinical basis for optimizing the management of patients with endometriosis and for early psychological intervention during the epidemic.

## Data Availability

All data generated or analyzed during this study are included in this published article.
